# Protective Microglia and Their Regulation in Parkinson’s Disease

**DOI:** 10.3389/fnmol.2016.00089

**Published:** 2016-09-21

**Authors:** Weidong Le, Junjiao Wu, Yu Tang

**Affiliations:** ^1^Center for Clinical Research on Neurological Diseases, First Affiliated Hospital, Dalian Medical University, DalianChina; ^2^Department of Rheumatology and Immunology, Xiangya Hospital of Central South University, ChangshaChina; ^3^Department of Molecular Biology, University of Texas Southwestern Medical Center, Dallas, TXUSA; ^4^Hamon Center for Regenerative Science and Medicine, University of Texas Southwestern Medical Center, Dallas, TXUSA

**Keywords:** Parkinson’s disease, neuroinflammation, anti-inflammation, transrepression pathway, histone modification, microRNA, alternative activation

## Abstract

Microglia-mediated neuroinflammation is a hallmark of Parkinson’s disease (PD). In the brains of patients with PD, microglia have both neurotoxic and neuroprotective effects, depending on their activation state. In this review, we focus on recent research demonstrating the neuroprotective role of microglia in PD. Accumulating evidence indicates that the protective mechanisms of microglia may result from their regulation of transrepression pathways via nuclear receptors, anti-inflammatory responses, neuron–microglia crosstalk, histone modification, and microRNA regulation. All of these mechanisms work together to suppress the production of neurotoxic inflammatory components. However, during the progression of PD, the detrimental effects of inflammation overpower the protective actions of microglia. Therefore, an in-depth exploration of the mechanisms underlying microglial neuroprotection, and a means of promoting the transformation of microglia to the protective phenotype, are urgently needed for the treatment of PD.

## Introduction

Parkinson’s disease (PD) is the second most common neurodegenerative disease, and manifests as a variety of movement defects (Dauer and Przedborski, 2003). The disease is characterized clinically by the loss of dopaminergic neurons in the substantia nigra of the midbrain, and pathologically by the accumulation of Lewy bodies (protein aggregates containing α-synuclein) in the remaining dopaminergic neurons (Braak et al., 2003). As the majority (>95%) of PD cases occur sporadically, the cause and pathogenesis of PD are now believed to be related to environmental factors alone or in combination with a genetic predisposition (Samii et al., 2004; Gao and Hong, 2011; Mullin and Schapira, 2015).

Extensive and consistent neuroinflammation is an important component in the pathogenesis of PD (Nagatsu and Sawada, 2005; Block and Hong, 2007). The involvement of neuroinflammation in dopaminergic neuron loss in PD is supported by a wealth of clinical and molecular evidence. For example, postmortem analysis of patients with PD revealed a large number of activated microglia and accumulation of inflammatory mediators in the substantia nigra (Hirsch and Hunot, 2009). Positron emission tomography also showed an increase in microglial activation in the early stages of PD that was correlated inversely with dopaminergic terminal density, and positively with motor impairment (Ouchi et al., 2005). In addition, cerebrospinal fluid from patients with PD is cytotoxic to dopaminergic neurons owing to the elevated levels of cytokines and antibodies which may interact with proteins modified by dopamine oxidation (He et al., 2002; Nagatsu and Sawada, 2005).

Whether neuroinflammation is the cause or consequence of dopaminergic neuron degeneration remains unknown, and how microglial activation produces detrimental or beneficial effects is not yet fully determined. The neuroprotective effects of microglia in various central nervous system (CNS) diseases including PD have drawn increasing attention in recent years. In this review, we will focus on the protective roles of microglia in PD and summarize new research that has uncovered the molecular mechanisms underlying the transformation of microglia to their neuroprotective phenotype.

## Neuroinflammation: Angel or Devil?

Neuroinflammation has been described extensively in PD, and is becoming recognized as a double-edged sword, producing both detrimental and beneficial effects (Sierra et al., 2013; Benraiss et al., 2016). Neuroinflammation is a complex integration of responses from all immune cells present within the CNS, including microglia, astrocytes, and infiltrating T-lymphocytes. Astrocytes are the most abundant glial cells of the nervous system and provide essential functional support for neurons, including antioxidant protection, glutamate clearance, promotion of neurovascular coupling, and release of transmitters and cytokines (Volterra and Meldolesi, 2005; Oberheim et al., 2012). However, in addition to their numerous protective functions already reported in PD (Rappold and Tieu, 2010; Mythri et al., 2011; Nam et al., 2015b), astrocytes also communicate with microglia to amplify the immune response and activate apoptotic mechanisms that induce dopaminergic neuronal death (Saijo et al., 2009). The small portion of infiltrated CD4^+^ T-lymphocytes that invade the substantia nigra also play an important role in mediating neuroinflammation in animal models of PD, albeit with divergent functions (Reynolds et al., 2007; Brochard et al., 2009).

Although microglia represent only 5–20% of the CNS cell population, they provide the first line of defense for the innate immune system against infection or injury (Nimmerjahn et al., 2005). Under physiological conditions, microglia exhibit a deactivated phenotype and constantly survey the microenvironment to maintain tissue homeostasis (Streit, 2002). They sense a wide range of stimuli through a combination of diverse membrane receptors, termed pattern recognition receptors, which are constitutively expressed to identify and bind pathogen- and damage-associated molecular patterns (Akira et al., 2006; Block et al., 2007; Tang and Le, 2014). The stimuli that may directly or indirectly lead to microglial activation, especially in the substantia nigra, generally derive from brain trauma, infection, cell debris, degraded neuromelanin, environmental toxins, and released protein aggregates (Davalos et al., 2005; Hanisch and Kettenmann, 2007). Microglia are often rapidly activated, changing morphology and secreting a spectrum of pro-inflammatory mediators to engulf infectious organisms or invading pathogens, and clearing toxic proteins and cell debris from the injury site by phagocytosis (Gao et al., 2003; Schwartz and Kipnis, 2004; Glezer et al., 2007; Gao and Hong, 2008).

Microglial activation also enhances neuronal survival by releasing trophic and anti-inflammatory factors (Ding et al., 2004; Schwartz and Kipnis, 2004). Indeed, glial-derived neurotrophic factor was shown to enhance neuronal survival and rescue injured dopaminergic neurons in animal models and in a clinical trial of gene therapy for PD (Kordower, 2003; Ding et al., 2004; Nam et al., 2015a). In addition to removing harmful stimuli, under certain circumstances microglial activation may enhance recovery and the healing process by promoting the expression of genes involved in tissue repair and regeneration (Schwartz and Kipnis, 2004; Glezer et al., 2007; Schwartz and Ziv, 2008). Therefore, without neuroinflammation, removal of offending pathogens and recovery from CNS injuries might be compromised.

However, in PD, the harmful molecules persistently released by microglia in the substantia nigra usually overshadow the beneficial molecules, so that the overall effect of microglial activation is detrimental. For example, reactive microglia in the nigrostriatal pathway can produce a large amount of pro-inflammatory cytokines, such as tumor necrosis factor-α (TNF-α), interleukin (IL)-1β and IL-6, and multiple chemokines, as well as superoxide and nitric oxide (NO), which may augment neuronal degeneration (Block et al., 2007; Dufek et al., 2015). In PD, midbrain dopaminergic neurons are especially vulnerable and extremely sensitive to cytokines, probably due to dopamine metabolism and a dense population of microglia in the substantia nigra (Kim et al., 2000; Obeso et al., 2010; Tansey and Goldberg, 2010). Therefore, during disease progression, those pro-inflammatory mediators together with the neuron debris induce, in turn, more widespread damage to neighboring neurons—a process known as reactive microgliosis. Consequently, a cycle of neuronal injury and sustained inflammation occurs (Block et al., 2007). To break this cycle, a large number of anti-inflammatory agents have been tested, such as non-steroidal anti-inflammatory drugs (Gao and Hong, 2008) and minocycline (He et al., 2001), with promising results in preclinical trials.

## Pathogenic Proteins and Microglial Activation

### α-Synuclein

In the development of PD, microglia can be activated directly or indirectly by a range of misfolded proteins or pathogens. For example, the mutated forms of α-synuclein, one of the most prevalent pathological proteins identified in familial PD, are generally aggregated, nitrated or oxidized, and released into the extracellular space from dying or dead dopaminergic neurons (Zhang et al., 2007; Gao et al., 2008; Reynolds et al., 2008; Lashuel et al., 2013). α-Synuclein aggregates act as chemoattractants to direct microglial migration toward damaged neurons through H_2_O_2_-dependent phosphorylation of tyrosine protein kinase Lyn (Wang et al., 2015), and consequently induce robust microglial activation by sensing Toll-like receptors (TLRs; Kim C. et al., 2013). Interestingly, both overexpression of mutant α-synuclein and a lack of α-synuclein in microglia can alter their immune profiles and phagocytic ability (Austin et al., 2006; Rojanathammanee et al., 2011), suggesting a potential autonomous microglial reaction in the PD models harboring α-synuclein mutations.

### Leucine-Rich Repeat Kinase 2

Leucine-rich repeat kinase 2 (LRRK2) is an autosomal dominant, late-onset familial PD gene. Its mutated form has recently been demonstrated to be a negative regulator of microglial motility, which thus prevents microglia from efficiently responding to brain damage (Choi et al., 2015). LRRK2 also plays a role in mediating microglial morphology and pro-inflammatory responses. Inhibition of LRRK2 kinase activity or knockdown of LRRK2 protein changes lipopolysaccharide (LPS)–TLR4-induced outgrowth of microglial process and attenuates the induction of cytokines such as inducible nitric oxide synthase (iNOS), TNF-α, IL-1β and IL-6 (Kim et al., 2012; Moehle et al., 2012). LPS-induced phosphorylation of p38 mitogen-activated protein kinase (MAPK) and stimulated NF-κB transcriptional activity is also decreased in LRRK2 knockdown cells, demonstrating that LRRK2 is a critical component in the mediation of neuroinflammation in PD (Kim et al., 2012).

### Parkin

Loss-of-function mutations in the gene encoding parkin, a ubiquitin E3 ligase protein, are responsible for autosomal recessive PD (Lucking et al., 2000). Although, most studies on parkin have focused on its function in neurons, its levels can also be regulated by inflammatory signaling in microglia treated with LPS or TNF-α (Tran et al., 2011). Parkin deficiency greatly increases the vulnerability of nigral dopaminergic neurons to inflammation-related degeneration in mice, with increased levels of TNF-α, IL-1β, and iNOS mRNA (Frank-Cannon et al., 2008; Tran et al., 2011). Notably, aged parkin-null mice display increased astrogliosis in the striatum and aberrant microglial activation in the midbrain (Rodriguez-Navarro et al., 2007). They also accumulate higher levels of tau and fail to upregulate heat shock proteins (Rodriguez-Navarro et al., 2007). Similarly, another study showed that aged parkin-null microglia produce markedly less glutathione and are more sensitive to H_2_O_2_-induced loss of viability than wild-type microglia of a similar age (Solano et al., 2008).

### DJ-1

Mutations in the gene encoding DJ-1, an oxidative stress sensor that localizes to mitochondria, have been linked to the development of early onset PD (Macedo et al., 2003). Knockdown of DJ-1 in microglia increases cell sensitivity to dopamine, measured by secreted pro-inflammatory cytokines such as IL-1β and IL-6 (Trudler et al., 2014). DJ-1-deficient microglia show elevated monoamine oxidase activity that induces a high level of intracellular reactive oxygen species and NO, leading to increased dopaminergic neurotoxicity (Trudler et al., 2014). Signal transducers and activators of transcription (STATs) are pivotal signaling molecules that activate neuroinflammation induced by interferon-γ (IFN-γ; Horvath, 2004). It is reported that microglia cultured from DJ-1-null mice express a high level of pro-inflammatory mediators and phosphorylated STAT1 (p-STAT1) in response to IFN-γ, and IFN-γ-induced interactions of Src-homology 2-domain containing protein tyrosine phosphatase-1 (SHP-1) with p-STAT1 and STAT1 are also attenuated (Kim J. H. et al., 2013). Direct intranigral LPS administration causes a greater loss of dopaminergic neurons and striatal dopamine content in DJ-1-null mice than in wild-types (Chien et al., 2016). Furthermore, LPS-induced neuronal death in neuron–glia co-cultures is augmented by DJ-1 deficiency in microglia, which can be antagonized by the neutralizing antibody against IFN-γ (Chien et al., 2016). Therefore, loss of DJ-1 function might increase the risk of PD by enhancing neuroinflammation.

### Matrix Metalloproteinases

Matrix metalloproteinases (MMPs) belong to a family of extracellular soluble or membrane-bound endopeptidases, which are mainly responsible for the remodeling of extracellular macromolecules (Lu et al., 2011). The activation of MMPs, particularly MMP-3 and MMP-9, might be associated with PD pathogenesis (Kim and Hwang, 2011; He et al., 2013). Expression of MMPs is elevated in various PD models established using selective toxins and inflammation (Lorenzl et al., 2002; Kim and Hwang, 2011). MMP-3 is induced and activated in dopaminergic neurons upon stress conditions, and its active form is then released into the medium (Kim et al., 2005; Kim et al., 2007). The released MMP-3 activates microglia, enhances the NF-κB signaling pathway, increases the TNF-α level and eventually causes neuronal death (Kim et al., 2005, 2007). In the 1-methyl-4-phenyl-1,2,3,6-tetrahydropyridine (MPTP)-injected PD model, dopaminergic neuron degeneration, microglial activation, and superoxide generation are largely attenuated in MMP-3-null mice compared to wild-types (Kim et al., 2007). Similarly, inhibition of MMP-9 with Ro 28-2653 can significantly reduce dopamine depletion and loss of dopaminergic neurons in the substantia nigra (Lorenzl et al., 2004). All results suggest that MMPs are critical in the immunopathogenesis of PD, and that MMP suppression might be a useful therapeutic strategy for PD.

## Activation States of Microglia

Microglial immune responses have been widely investigated and demonstrated to be significant heterogeneous and show distinct region-dependent diversity (Hanisch, 2013; Grabert et al., 2016). Microglial activation states, or phenotypes, have been increasingly studied in recent years. To simplify their functional heterogeneity, microglia are polarized into two contrary states termed classical activation state and alternative activation state depending on the types of stimuli, echoing the divergent effects of neuroinflammation (Colton, 2009; Colton and Wilcock, 2010). Classical activation of microglia has been widely studied and is likely to be the more common response. In this state, microglia are usually induced by multiple pathogen- and damage-associated molecular patterns, and produce pro-inflammatory cytokines, reactive oxygen species, NO and superoxide (Block et al., 2007). In contrast, alternative activation refers to a state that promotes the expression of genes involved in inflammation resolution, tissue repair, and extracellular matrix reconstruction, such as arginase 1 (*Arg1*), mannose receptor (*CD206*), found in inflammatory zone 1 (*Fizz1* or *Retnla*), and chitinase 3-like 3 (*Chi3l3* or *Ym1*) (Colton, 2009). Microglia are assumed to transition between the two activation states both in normal tissue and under pathogenic conditions. Imbalance between the two reactive phenotypes has been attributed to the development and progression of PD (Tang and Le, 2016). However, this view of microglial categorization has been challenged in light of new research findings and technological advances. With the advent of single-cell RNA sequencing, studies have also recently shown that the cells such as microglia/macrophages, express classical and alternative activation simultaneously in response to traumatic brain injury, resulted from mixed populations or from lack of activation signatures in individual cells (Kim et al., 2016; Morganti et al., 2016). This hints that solely activation states might not exist, and expression of polarization markers might be ineffective in predicting the presence of other polarization genes (Ransohoff, 2016). Nevertheless, enhancement of alternative activation-like responses has been demonstrated to partly contribute to neuroprotection. In the remainder of this review, we will summarize recent findings on the various protective mechanisms of microglia and meanwhile list their links with different microglial states.

## Protective Mechanisms of Microglia

**FIGURE 1:**
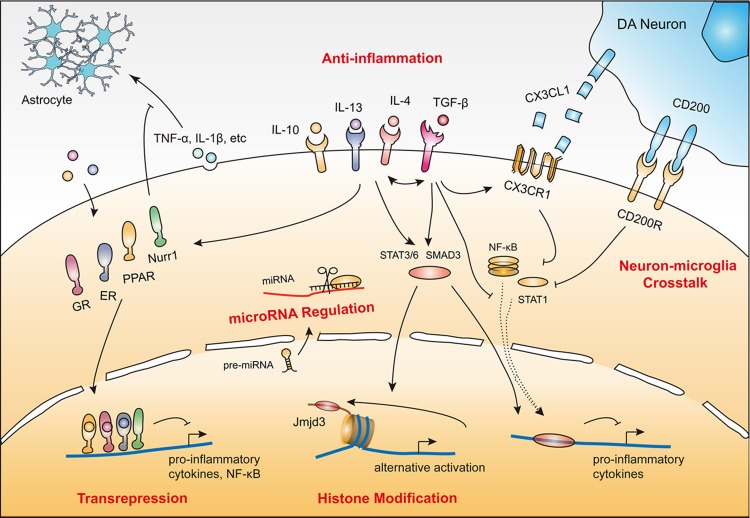
**Protective mechanisms of microglia in PD.** (1) Anti-inflammatory action induced by cytokines (e.g., IL-4, IL-10, IL-13 and TGF-β). (2) Transrepression pathways through multiple nuclear receptors (e.g., GRs, PPARs, ERs, and Nurr1). (3) Neuron–microglia crosstalk (CD200–CD200R and CX3CL1–CX3CR1). (4) Histone modification (e.g., JMJD3). (5) MicroRNA regulation (e.g., miR-124). These protective mechanisms coordinate with each other to render microglia immunosuppressive or quiescent by inhibiting neurotoxic inflammatory components including reactive oxygen species, nitric oxide (NO), superoxide, various pro-inflammatory cytokines, and NF-κB and its downstream targets.

Given the vulnerability and poor regenerative capacity of dopamine neurons, sustained inflammation could drive a chronic neurodegenerative process. Fortunately, endogenous protective regulatory signals and negative feedback mechanisms attenuate microglial neurotoxicity. This inhibitory feedback is critical in both intact and injured tissue. According to the current state of knowledge, microglial protective mechanisms relevant to PD pathology generally involve: (1) anti-inflammation; (2) transrepression pathways through multiple nuclear receptors; (3) neuron–microglia crosstalk; (4) histone modification; and (5) microRNA regulation (**Table 1**). All these mechanisms converge to inhibit pro-inflammatory cytokines, reactive oxygen species, and NO production, and suppress the activity of NF-κB and its downstream targets (**Figure 1**).

**Table 1:** Neuroprotective mechanisms of microglia-mediated neuroinflammation.

	Typical mechanisms	In crosstalk with	Associated with alternative activation
**Anti-inflammation**			
IL-4 IL-13	(1) Suppress the pro-inflammation (2) Reduce superoxide and nitrite production (3) Assist the inflammation resolution and tissue repair	TGF-β, PPARs, CD200, JMJD3 miR-124	Yes
IL-10	(1) Suppresses the pro-inflammation		Yes
TGF-β	(1) Suppresses the pro-inflammation (2) Increases ECM deposition (3) Promotes tissue repair	IL-4, CX3CR1	Yes
**Transrepression pathways**			
GRs	(1) Suppress the NF-κB activity (2) Inhibit pro-inflammatory mediators (3) Reduce nitrite production		
PPARs	(1) Repress NF-κB and MAPK activities (2) Inhibit pro-inflammatory cytokines (3) Reduce nitrite production	IL-4 IL-13	Yes
ERs	(1) Inhibit pro-inflammatory cytokines (2) Activate the PI3K pathway (3) Suppress the NF-κB activity (4) Inhibit inwardly rectifying K^+^ channel Kir2.1		
Nurr1	(1) Interacts with CoREST (2) Inhibits pro-inflammatory cytokines (3) Suppresses the NF-κB activity		Yes
**Neuron-microglia crosstalk**			
CD200-CD200R	(1) Promotes the KATP channels open (2) Inhibits the ATP release (3) Inhibits pro-inflammatory cytokines (4) Suppresses the iNOS activity	IL-4	
CX3CL1-CX3CR1	(1) Inhibits pro-inflammatory cytokines (2) Reduces nitrite production	TGF-β	Yes
**Histone modification**			
JMJD3	(1) Inhibits pro-inflammatory cytokines (2) Reduces nitrite production (3) Suppresses the NF-κB activity	IL-4	Yes
**MicroRNA regulation**			
miR-124	(1) Inhibits classical activation (2) Promotes alternative activation		Yes
miR-21 miR-181c	(1) Suppress Fas ligand or TNF-α production		
miR-155	(1) Inhibition of miR-155 switches microglial activation toward alternative activation		Yes

*CX3CL1, fractalkine; CX3CR1, fractalkine receptor; ECM, extracellular matrix; ER, estrogen receptor; GR, glucocorticoid receptor; IL, interleukin; PPAR, peroxisome proliferator-activated receptor; TGF-β, transforming growth factor-β.*

### Anti-inflammation

IL-4, IL-13, IL-10 and transforming growth factor-β (TGF-β) are the major anti-inflammatory cytokines that play a critical role in minimizing brain inflammation and enhancing the expression of genes involved in tissue recovery (Colton and Wilcock, 2010; Saijo and Glass, 2011). Of these, IL-4 and IL-13 are well-described. They are recognized by IL-4/IL-13 receptors to suppress the production of pro-inflammatory cytokines such as IL-6, IL-8 and TNF-α and reduce superoxide production and NO release, which ultimately alleviates LPS-induced neuron injury both *in vitro* and *in vivo* (Ledeboer et al., 2000; Butovsky et al., 2005; Park et al., 2005; Zhao et al., 2006; Colton, 2009; Colton and Wilcock, 2010; Saijo and Glass, 2011). IL-4 secreted from neurons also enhances microglial expression of the IL-4 receptor, facilitating a “feedforward” increase in microglial expression of trophic factors, and peroxisome proliferator-activated receptor (PPAR)-dependent phagocytosis of apoptotic neurons (Zhao et al., 2015). Similarly, in LPS-induced mouse model of PD, intracerebral IL-10 alleviated microglial activation and inhibited LPS-mediated production of TNF-α and NO, subsequently leading to neuroprotection against LPS-induced dopaminergic neuronal death (Rentzos et al., 2009). However, these protective effects of IL-10 are not seen in mice that lack nicotinamide adenine dinucleotide phosphate (NADPH) oxidase (Arimoto et al., 2007), suggesting that IL-10 might antagonize NADPH oxidase to promote anti-inflammation. TGF-β is a pleiotropic cytokine with diverse functions including the induction of angiogenesis and promotion of extracellular matrix deposition, and it also participates in suppressing microglial responses, thus avoiding exacerbation of brain damage after injury (Boche et al., 2006). It should be noted that different anti-inflammatory cytokines (IL-4, IL-10, TGF-β) might differentially affect the production of pro-inflammatory mediators (Ledeboer et al., 2000).

Anti-inflammatory cytokines are also powerful triggers of alternative activation in microglia, and aid inflammation resolution through endocytic clearance, promote tissue repair and, in turn, induce the secretion of higher levels of IL-4, IL-10, and IGF-1 to enhance neuronal survival (Ponomarev et al., 2007; Colton, 2009; Henkel et al., 2009; Colton and Wilcock, 2010; Zhou et al., 2012). IL-4 production, for example, is essential for the maintenance of *Ym1* expression and alternative activation in microglia and peripheral infiltrating macrophages (Ponomarev et al., 2007).

### Transrepression Pathways

#### Glucocorticoid Receptors

Glucocorticoids are the most efficient endocrine molecules sensed by glucocorticoid receptors (GRs) and have anti-inflammatory and immunosuppressive effects (Herrero et al., 2015; Xavier et al., 2016). Upon binding, GRs translocate into the nucleus, resulting in the suppression of NF-κB activity and downregulation of a variety of pro-inflammatory mediators (Nelson et al., 2003). GRs deficiency induces persistent microglial activation with higher nitrite production that precedes the loss of dopaminergic neurons (Morale et al., 2004; Ros-Bernal et al., 2011). This excitotoxicity can be reduced by the GR agonist dexamethasone and increased by the GR antagonist RU486 (Gallina et al., 2015). GR-deficient microglia also have higher levels of pro-inflammatory cytokines (e.g., TNF-α) and inhibit the expression of anti-inflammatory genes (e.g., IL-1R2; Ros-Bernal et al., 2011). In GR-deficient mice, intraparenchymal injection of LPS activates the TLR4 signaling pathway, resulting in exacerbated cellular lesions and neuronal and axonal damage (Carrillo-de Sauvage et al., 2013). Interestingly, in the MPTP-intoxicated mouse model and in patients with PD, GRs expression is reduced in the substantia nigra, suggesting that the neuroprotective effects of GRs might be impaired in PD pathogenesis (Ros-Bernal et al., 2011).

#### Peroxisome Proliferator-Activated Receptors

Peroxisome proliferator-activated receptors belong to the steroid hormone nuclear receptor superfamily, which has been extensively studied with respect to its regulation of glucose and lipid metabolism, energy balance, atherosclerosis, and macrophage differentiation. PPARs also play a pivotal role in inflammation regulation in the CNS through transrepression pathways (Breidert et al., 2002; Dehmer et al., 2004; Hunter et al., 2007; Quinn et al., 2008).

Activation of PPARs inhibits the synthesis of pro-inflammatory mediators including TNF-α, NO, cyclooxygenase-2 and related chemokines, to attenuate neurotoxicity (Bernardo et al., 2000; Kim et al., 2002). For example, treatment with 15-deoxy-Δ^12-14^-prostaglandin J2, one of the major natural PPAR-γ agonists, inhibits LPS-induced microglial activation (Bernardo et al., 2000). Similarly, synthetic agonists of PPAR-γ, including troglitazone, ciglitazone and pioglitazone, can also suppress the excessive production of pro-inflammatory molecules and prevent LPS-induced neuronal death (Kim et al., 2002; Hunter et al., 2007). Inhibition of microglial activation by PPARβ/δ is also associated with the suppression of NF-κB and MAPK activity (Xu et al., 2013).

Malibatol A, a novel natural antioxidant extracted from the Chinese plant *Hopea hainanensis*, inhibits inflammatory cytokines not only in LPS-stimulated microglia but also in mouse models of stroke (Pan et al., 2015). Treatment with malibatol A decreases classical activation markers (CD16, CD32, and CD86) and increases alternative activation markers (CD206, Ym1) by activating PPAR-γ (Pan et al., 2015). In the MPTP-injected mouse model of PD, pioglitazone administration attenuates dopaminergic neuron death by blocking the NF-κB pathway and inhibiting iNOS (Breidert et al., 2002; Dehmer et al., 2004; Quinn et al., 2008). Similarly, oral treatment with telmisartan, the most potent PPAR-γ activator, provides neuroprotection against dopaminergic cell death and neuroinflammation in MPTP-lesioned mice, which can be inhibited by co-administration of the PPAR-γ antagonist GW9662 (Garrido-Gil et al., 2012). Together, these studies demonstrate the potential of PPARs as targets for resolving neuroinflammation and attenuating neurotoxicity.

#### Estrogen Receptors

Epidemiological studies have found a higher prevalence of PD in men than in women, and drugs used to treat PD affect men and women differently with regard to therapeutic responses (Shulman, 2002; Tao et al., 2012). Different levels of steroid hormones such as estrogens are believed to be an important contributor to these differences. The anti-inflammatory effects of estrogens and estrogen receptor (ER)-selective ligands on microglial activation have been extensively evaluated. Generally, estrogens act through ERα or ERβ receptors to suppress the production of several proinflammatory cytokines, including TNF-α and other secretory products induced by LPS, to protect neurons against death (Vegeto et al., 2000, 2001; Liu et al., 2005). Estrogen also ameliorates microglial activation by inhibiting the inwardly rectifying K^+^ channel Kir2.1, a known regulator of microglial activation (Wu et al., 2016). Notably, those protective effects can be blocked by ER antagonists, suggesting that ERs are critical mediators of the anti-inflammatory and neuroprotective functions of estrogens (Vegeto et al., 2001; Liu et al., 2005; Ishihara et al., 2015). Specifically, ERα exerts its anti-inflammatory effect via the PI3K pathway, which in turn blocks the NF-κB pathway and translocation to the cell nucleus (Ghisletti et al., 2005). Moreover, estrogens also suppress microglial activation and attenuate the loss of dopaminergic neurons in MPTP-intoxicated male mice *in vivo* (Tripanichkul et al., 2006). Collectively, this evidence highlights the importance of ERs in the inhibitory effects of estrogens that might account for the gender differences in PD.

#### Nurr1

Nurr1 (NR4A2) is a member of the NR4A subfamily of orphan nuclear receptors. It is required for the differentiation and maintenance of midbrain dopaminergic neurons and is an important pathogenic gene for familial PD (Le et al., 2003; Jankovic et al., 2005; Kadkhodaei et al., 2009). The transrepression effects of Nurr1 on neuroinflammation have gained increasing attention in recent years. Nurr1 expression is increased in microglia responding to LPS stimulation and the protein is translocated from the cytoplasm to the nucleus to regulate gene expression (Fan et al., 2009). In the LPS-injected PD model, Nurr1 mediates transrepression pathways (Saijo et al., 2009). Overexpressed Nurr1, orchestrated with Corepressor for Repressor Element 1 Silencing Transcription Factor (CoREST) inhibits the expression of various pro-inflammatory neurotoxic molecules and NF-κB targeted genes. In contrast, knockdown of Nurr1 gives rise to exaggerated inflammatory responses in microglia that are further amplified by astrocytes, causing the extensive death of dopaminergic neurons (Saijo et al., 2009). These studies reveal Nurr1 as a novel pharmacological target in PD immunotherapy.

Nurr1 is an orphan nuclear receptor which acts by ligand-independent activation. However, Nurr1 agonists such as 1,1-bis (3′-indolyl)-1-(*p*-chlorophenyl) methane (C-DIM12) and SA00025 have been generated in recent years and stabilize binding of CoREST, block pro-inflammatory gene expression by inhibiting NF-κB, induce dopaminergic gene expression, and thus produce anti-parkinsonian effects against 6-hydroxydopamine intoxication (Zhang et al., 2012c,d; De Miranda et al., 2015; Hammond et al., 2015; Smith et al., 2015). Forced expression of Nurr1 and forkhead box A2 (Foxa2), a potent Nurr1 co-activator in the development of dopaminergic neurons (Yi et al., 2014), causes microglial phenotypes to switch, and synergistically protects degenerating dopaminergic neurons by paracrine signaling (Oh et al., 2015). Similarly, exogenous expression of Nurr1 transcriptionally activates the expression of alternative activation markers such as Arg1, by directly binding to its promoter (Mahajan et al., 2015). Overall, Nurr1 might function as an anti-inflammatory transcription factor and contribute to the balance of different microglial phenotypes.

### Neuron–Microglia Crosstalk

#### CD200 and its Receptor (CD200R)

CD200R is present on microglia, and actively drives quiescent microglia by engaging CD200, a type I membrane glycoprotein expressed on the membrane surface of neurons (Vieites et al., 2003; Gorczynski et al., 2004; Lyons et al., 2007). Microglia in CD200-deficient mice exhibit more characteristics of activation than in wild-types, appearing aggregated, less ramified, and with shorter glial processes (Hoek et al., 2000; Deckert et al., 2006). Moreover, the increased microglial activation in CD200-deficient mice is accompanied by elevated levels of TNF-α and iNOS, suggesting that neuronal inhibitory signals for microglial response are compromised (Deckert et al., 2006). Disruption of the CD200–CD200R interaction by a CD200R antibody greatly exacerbates dopaminergic neuron death in a primary neuron/microglia co-culture system (Wang et al., 2011).

There is also a time-dependent downregulation of CD200–CD200R in the cerebra of MPTP-injected mouse models of PD (Ren et al., 2016). CD200 appears to promote the opening of the adenosine triphosphate-sensitive potassium (KATP) channels, inhibit microglial activation and the release of ATP and pro-inflammatory factors, and protect dopaminergic neurons against MPTP-induced lesions (Ren et al., 2016). Furthermore, blocking CD200–CD200R interaction exaggerates microglial activation with elevated TNF-α and IL-6 production in a 6-hydroxydopamine-induced rat model of PD, eventually causing more extensive dopaminergic neuron loss (Zhang et al., 2011).

Interestingly, CD200 expression is decreased in neurons from IL-4-deficient mice, and this induces a greater neuroinflammatory response to LPS (Lyons et al., 2009). Conversely, stimulation with IL-4 enhances CD200 expression, revealing a putative role of CD200 and CD200R in the alternative activation of microglia (Lyons et al., 2009; Yi et al., 2012). Together, this evidence suggests that the CD200–CD200R pathway is critical in the attenuation of microglial activation. Impairment in either CD200 or CD200R induces activation of microglia. We can also imagine that in the substantia nigra of patients with PD, the progressive loss of dopaminergic neurons cannot produce enough inhibitory CD200 that might accelerate reactive microgliosis and neurodegeneration.

#### Fractalkine (CX3CL1) and its Receptor (CX3CR1)

CX3CL1 is a transmembrane glycoprotein. It is highly expressed in neurons and cleaved from membranes in response to neurotoxic insults, attracting reactive immune cells such as microglia by binding with CX3CR1 (Chapman et al., 2000; Cook et al., 2001). The interaction between CX3CL1 and CX3CR1 attenuates microglial activation and neurodegeneration. For example, treatment with CX3CL1 suppresses the production of NO, IL-6 and TNF-α released from microglia upon LPS/IFN-γ stimulation that significantly prevents neuronal death *in vitro* (Mizuno et al., 2003). Cardona et al. (2006) used several *in vivo* models to demonstrate that a deficiency in CX3CR1 dysregulates microglial responses and results in neurotoxicity. CX3CR1-null mice showed increased cell-autonomous microglial activation and enhanced neurotoxicity after LPS-induced systemic inflammation (Cardona et al., 2006). This was also seen in an MPTP-intoxicated CX3CR1-null mouse model (Cardona et al., 2006), suggesting that CX3CR1 signaling is important in protecting neurons against microglial neurotoxicity. Therefore, augmenting neuroprotective CX3CL1–CX3CR1 signaling may be another avenue to investigate in the treatment of PD.

### Histone Modification

Alternatively activated microglia may facilitate neuroprotection and enhance tissue repair. It is therefore very important to probe the mechanisms that regulate alternative activation. Epigenetic changes, such as DNA methylation or histone structure alterations, regulate gene transcription by controlling the accessibility of the promoter to regulatory factors (Berger, 2007).

It has been reported that the histone H3K27me3 demethylase JMJD3 is essential for alternative microglial activation (Tang et al., 2014). Knockdown of JMJD3 compromises the expression of Arg1 and CD206 in IL-4 treated microglia, but exaggerates the production of pro-inflammatory cytokines and NO, eventually accelerating death of dopaminergic neurons *in vitro* (Tang et al., 2014). Arg1, a classical marker of alternative activation, is directly regulated by JMJD3 and shown to be anti-inflammatory by counteracting iNOS (Tang et al., 2014). This suggests that switching microglial phenotype is possible through epigenetic modification. Notably, this switch has also been studied in other neurodegenerative diseases, by regulating pivotal genes including *MSX3* (Yu et al., 2015), *FAM19A3* (Shao et al., 2015) and *NF-κB p50* (Taetzsch et al., 2015), or chemical treatments including fasudil (Zhang et al., 2013), malibatol A (Pan et al., 2015), and glatiramer acetate (Burger et al., 2009; Begum-Haque et al., 2013). Considering that microglial phenotypes can be further divided into various subtypes, probably with different effects on neuroprotection, more investigations into the detailed mechanisms involved in this switch are necessary.

### MicroRNA Regulation

MicroRNAs participate in the promotion of microglial quiescence. Broadly speaking, microRNA regulation is also an epigenetic mechanism. Results of microarray expression profiling and bioinformatics analysis of mRNA and microRNA from primary cultures of murine microglia showed that miR-689, miR-124, and miR-155 were the most strongly associated microRNAs predicted to mediate pro-inflammatory pathways and the classical activation phenotype (Freilich et al., 2013).

miR-124 is a modulator of microglia and macrophage activation recently demonstrated to maintain their quiescent state (Ponomarev et al., 2007, 2011). Knockdown of miR-124 leads to activation of microglia and peripheral macrophages *in vitro* and *in vivo*. Administration of miR-124 before or after disease onset causes systemic deactivation and suppression of experimental autoimmune encephalomyelitis symptoms (Ponomarev et al., 2011). More interestingly, transfection of miR-124 can attenuate the expression of markers associated with classical activation such as CD86 and iNOS, whereas cytokines and markers associated with alternative activation including Arg1, FIZZ1, and TGF-β are upregulated (Ponomarev et al., 2011). This finding is in line with the notion that quiescent microglia show properties of the alternatively activated state. Notably, miR-124 levels decrease over time in the substantia nigra of the MPTP-induced mouse model of PD, suggesting that miR-124 depletion is involved in the pathogenesis of the disease (Kanagaraj et al., 2014).

In addition, miR-21 and miR-181c are inversely correlated with Fas ligand and TNF-α, respectively, during hypoxia-induced microglial activation (Zhang et al., 2012a,b). The ectopic expression of miR-21 or miR-181c suppresses Fas ligand or TNF-α production by directly binding to its 3′-untranslated region, thereby partially protecting neurons from death (Zhang et al., 2012b,a).

Besides the protective microRNAs, miR-155 appears to be a pro-inflammatory microRNA that can transform the microglial activation state to a classical-like phenotype. Specifically, miR-155 targets anti-inflammatory proteins as well as some alternative activation-associated genes—such as that encoding SMAD2, a transcription factor critical in the expression of Arg1, CD206 and IL-10—leading to the upregulation of several pro-inflammatory mediators characteristic of the classical phenotype including iNOS, IL-6 and TNF-α (Ruffell et al., 2009; Louafi et al., 2010; Cardoso et al., 2012).

## Summary and Perspectives

In conclusion, microglia employ a series of neuroprotective actions by activating multiple receptors, releasing anti-inflammatory cytokines, initiating neuron–microglia crosstalk, regulating microRNA, and modifying histone tails to suppress the expression of neurotoxic genes. Notably, these actions work together against inflammation-mediated neuronal injury (**Figure 1**). For example, TGF-β can enhance IL-4-induced alternatively activated microglia by increasing the expression of Arg1 and Ym1, whereas treatment with IL-4 can increase the level of TGF-β2, suggesting that TGF-β and IL-4 communicate with each other to promote protective microglia. Moreover, TGF-β can also enhance the steady state level of CX3CR1 that may help microglia to adhere to neurons (Chen et al., 2002). Another example lies in the relationship between IL-4 and PPARs. IL-4 can induce PPAR activation, whereas PPAR agonists can also increase IL-4 expression and attenuate the LPS-induced increase in major histocompatibility complex class II and IL-1β levels in microglia (Loane et al., 2009).

The available evidence strongly supports a link between neuroprotective mechanisms and the alternative activation state of microglia (**Table 1**). Alternatively activated microglia enhance the expression of anti-inflammatory cytokines and various transrepression receptors to execute neuroprotective functions. Anti-inflammatory cytokines such as IL-4 and IL-13, as well as PPAR-γ, miR-124 and JMJD3, promote alternative activation of microglia. It might be interesting to determine whether transrepression receptors such as GRs and ERs also contribute to alternative activation.

Neuroprotective mechanisms of microglia are constantly competing with neurotoxic signaling that is critical for the maintenance of homeostasis. In most cases, the inflammatory responses are self-limiting and finely regulated to keep the balance between pro-inflammation/injury and the resolution of inflammation/recovery. In PD, however, persistent stimuli, derived from endogenous factors—especially α-synuclein aggregates or the ubiquitin–proteasome system—and environmental cues, contribute to excessive inflammation (Gao and Hong, 2008). The detrimental effects thus gradually overpower the protective effects of microglia, and the sustained and uncontrolled inflammation, acting either as an initiator or as a secondary propagator, drives the chronic and progressive neurodegeneration observed in PD.

There remains an urgent need for a comprehensive understanding of the neuroprotective role of microglia. Instead of targeting any one of a large number of pro-inflammatory factors, enhancing the neuroprotective effects of microglia might be a more effective therapeutic strategy.

## Author Contributions

YT conceived the study and drafted the manuscript; YT, JW, and WL provided the discussion and critically revised the manuscript; YT prepared the tables and figure. All authors read and approved the final version of the manuscript.

## Conflict of Interest Statement

The authors declare that the research was conducted in the absence of any commercial or financial relationships that could be construed as a potential conflict of interest.
